# A loss-of-function variant in *SSFA2* causes male infertility with globozoospermia and failed oocyte activation

**DOI:** 10.1186/s12958-022-00976-5

**Published:** 2022-07-14

**Authors:** Gelin Huang, Xueguang Zhang, Guanping Yao, Lin Huang, Sixian Wu, Xiaoliang Li, Juncen Guo, Yuting Wen, Yan Wang, Lijun Shang, Na Li, Wenming Xu

**Affiliations:** 1 Department of Obstetrics/Gynecology, Key Laboratory of Obstetric, Gynecologic and Pediatric Diseases and Birth Defects of Ministry of Education, Joint Lab for Reproductive Medicine(SCU-CUHK), West China Second University Hospital, Sichuan University, Chengdu, China; 2 Department of Reproductive Medicine Center, The Affiliated Hospital of Zunyi Medical University, Zunyi, China; 3 Department of Reproductive Endocrinology of West China Second University Hospital, Key Laboratory of Obstetric, Gynecologic and Pediatric Diseases and Birth Defects of Ministry of Education, Sichuan University, Chengdu, China; 4 School of Human Sciences, London Metropolitan University, London, UK; 5 Laboratory of Medical Systems Biology, Guangzhou Women and Children’s Medical Center, Guangzhou Medical University, Guangzhou, China

**Keywords:** *SSFA2*, Globozoospermia, Male infertility, AOA-ICSI, Oocyte activation failure

## Abstract

Globozoospermia (OMIM: 102530) is a rare type of teratozoospermia (< 0.1%). The etiology of globozoospermia is complicated and has not been fully revealed. Here, we report an infertile patient with globozoospermia. Variational analysis revealed a homozygous missense variant in the *SSFA2* gene (NM_001130445.3: c.3671G > A; p.R1224Q) in the patient. This variant significantly reduced the protein expression of SSFA2. Immunofluorescence staining showed positive SSFA2 expression in the acrosome of human sperm. Liquid chromatography–mass spectrometry/mass spectrometry (LC–MS/MS) and Coimmunoprecipitation (Co-IP) analyses identified that GSTM3 and Actin interact with SSFA2. Further investigation revealed that for the patient, regular intracytoplasmic sperm injection (ICSI) treatment had a poor prognosis. However, Artificial oocyte activation (AOA) by a calcium ionophore (A23187) after ICSI successfully rescued the oocyte activation failure for the patient with the *SSFA2* variant, and the couple achieved a live birth. This study revealed that SSFA2 plays an important role in acrosome formation, and the homozygous c.3671G > A loss-of-function variant in *SSFA2* caused globozoospermia. *SSFA2* may represent a new gene in the genetic diagnosis of globozoospermia, especially the successful outcome of AOA-ICSI treatment for couples, which has potential value for clinicians in their treatment regimen selections.

**Supplementary Information:**

The online version contains supplementary material available at 10.1186/s12958-022-00976-5.

## Introduction

Infertility, defined by the World Health Organization (WHO) as the failure to achieve a pregnancy after 12 months or more of regular unprotected sexual intercourse, is a major concern in public health and affects approximately 8-12% of couples worldwide [1, 2]. Approximately 20-70% of affected couples are affected by male factors, and among these male factors, 40–60% remain unexplained owing to a multifactorial pathological condition [3, 4]. Genetic factors account for at least 15% of male infertility cases [5]. In recent years, with the widespread application of high-throughput sequencing technology, an increasing number of genetic factors leading to male infertility have been discovered. Despite such efforts, most genetic causes of human infertility are currently uncharacterized, and the discovery of novel genetic factors in idiopathic male infertility is a major challenge.

Globozoospermia (OMIM: 102530) is a rare type of teratozoospermia (< 0.1%), characterized by round-headed spermatozoa without acrosomes, and globozoospermia can be classified into total globozoospermia (type I) and partial globozoospermia (type II) [6, 7]. Previous studies have suggested that gene variants might be the pathology underlying human globozoospermia. To date, variants in several genes (*DPY19L2*, *PICK1*, *SPATA16*, *ZPBP*, *CCDC62*, *SPINK2*, *C2CD6*, *CCIN*, *C7orf61*, *DNAH17*, *GGN*, and *SPACA1*) have been identified as causing some human globozoospermia cases [8–13]. However, the known genetic defects can explain only approximately 75% of the cases of globozoospermia [11], and genetic causality remains unknown in the remaining patients.

In the present study, we identified a novel homozygous variant of c.3671G > A in sperm specific antigen 2 (*SSFA2*) in a globozoospermia patient from a consanguineous family by whole-exome sequencing (WES). This variant was not found in any of the 220 healthy controls. This gene encodes the protein of SSFA2 known as KRAP, which can tether IP3 receptors (IP3Rs) to Actin alongside sites and license IP3Rs to evoke Ca^2+^ puffs [14–16]. The negative effect of the variant on *SSFA2* expression and further sperm head morphology was confirmed by bioinformatic analysis and in vitro experiments. Using liquid chromatography–mass spectrometry/mass spectrometry (LC–MS/MS) analysis, we further confirmed that SSFA2 interacts with Actin and GSTM3 to maintain sperm head formation during spermatogenesis. Moreover, regular intracytoplasmic sperm injection (ICSI) was carried out on the patient, but SSFA2 and PLCζ deficiency resulted in oocyte activation failure and poor prognosis. Next, Artificial oocyte activation (AOA) by a calcium ionophore (A23187) after ICSI was applied to this patient in the second cycle and successfully overcame the oocyte activation failure. The couple obtained a healthy live birth. Together, we elucidated a novel variant in *SSFA2* causing globozoospermia, and ICSI with AOA may overcome infertility involving *SSFA2* variants.

## Materials and methods

### Study participants

A patient with primary infertility and his family were recruited from West China Second University Hospital, Sichuan University. His 26-year-old wife with normal ovulatory cycles was also recruited for ICSI treatment. A total of 220 healthy Chinese volunteers, as healthy controls, who had medical check-ups without evidence of any infertility were obtained from the Physical Examination Center in our hospital. This study was conducted following the tenets of the Declaration of Helsinki, and ethical approval was obtained from the Ethical Review Board of West China Second University Hospital, Sichuan University. All subjects signed an informed consent form.

### Whole-exome sequencing (WES) and Sanger sequencing

Peripheral blood samples were obtained from all subjects, and the genomic DNA was isolated by DNeasy Blood & Tissue Kits (69,504, QIAGEN) according to the manufacturer’s protocol. Next-generation sequencing was carried out using the SureSelectXT Human All Exon Kit (5190-8864, Agilent) and Illumina HiSeq X-TEN. The reads were mapped to the human reference sequence (UCSC hg19) using BWA 0.7.9a from the BWA-MEM algorithm. After quality filtering by the Genome Analysis Toolkit [17], functional annotation was performed using ANNOVAR through a series of databases, including the 1000Genomes Project, gnomAD, HGMD and ExAC. Next, PolyPhen-2, SIFT, MutationTaster and CADD were used for functional prediction. The *SSFA2* variant identified by WES was confirmed by Sanger sequencing. The PCR primers were as follows: F 5′ GCATCGGTGGCTCTAACGCCAACAG 3′; R 5′ TGGGACTACAGGCACATGCCACCAC 3′.

### Electron microscopy

For scanning electron microscopy (SEM), the sperm cells were fixed onto slides using 2.5% glutaraldehyde and refrigerated overnight at 4 °C. After rinsing the slides with 1 × PBS buffer three times, the slides were gradually dehydrated with an ethanol gradient (30, 50, 75, 95, and 100% ethanol) and dried by a CO_2_ critical-point dryer. After metal spraying by an ionic sprayer meter (Eiko E-1020, Hitachi), the samples were observed by SEM (S-3400, Hitachi).

For transmission electron microscopy (TEM), the sperm cells were washed with SpermRinse™ (10,101, Vitrolife) three times, fixed in 3% glutaraldehyde, phosphate-buffered to pH 7.4 and postfixed with 1% OsO4. After embedding in Epon 812, ultrathin sections were stained with uranyl acetate and lead citrate and observed under a TEM (TECNAI G2 F20, Philips) with an accelerating voltage of 120 kV.

### Immunofluorescence microscopy

Spermatogenic cells were coated on the slides and fixed in 4% paraformaldehyde for 15 min. Then, they were permeabilized with 3% bovine serum albumin (A1933, Sigma–Aldrich) and 0.1% Triton X-100 for 30 min at room temperature. Next, the samples were incubated overnight at 4 °C with primary antibodies against SSFA2 (1:200; 14,157-1-AP, Proteintech), GSTM3 (1:100; 67,634-1-Ig, Proteintech), F-Actin (1:500; ab205, Abcam), PLCζ (1:200; A65778-050, EpiGentek) and Peanut agglutinin (PNA) conjugated AlexaFluor 488 (1:100; L21409, Thermo Fisher Scientific); After washing with 1 × PBS buffer twice, the samples were incubated for 1 hour with Alexa Fluor 488 (1:1000; A21206, Thermo Fisher Scientific)- or Alexa Fluor 594 (1:1000; A11005, Thermo Fisher Scientific)-labeled secondary antibodies at room temperature; Nuclei were counterstained with 4′,6-diamidino-2-phenylindole (DAPI) (D9542, Sigma–Aldrich).

For the staining of testicular tissues, samples were fixed in 3.7% buffered formaldehyde. After fixation, the tissues were first embedded in paraffin. The samples were sectioned at a thickness of 5 μm. After deparaffinization and rehydration, the sections were treated with 3% hydrogen peroxide for 10 min at room temperature and with 20 mM sodium citrate for 15 min at 95 °C. Subsequently, after being washed twice with 1 × PBS buffer for 5 minutes, the sections were blocked with goat serum (16,210,072, Thermo Fisher Scientific) at 37 °C for 1 hour and then incubated with primary antibodies overnight at 4 °C, followed by 1 hour of incubation at 37 °C with secondary antibodies and 0.5% DAPI. Images were captured with a confocal microscope (Olympus FV3000).

### Cell culture and plasmid construction

In our study, HEK293T cells were obtained from the American Type Culture Collection (ATCC® CRL-11268™). HEK293T cells were grown in DMEM (11965092, Gibco) supplemented with 10% fetal bovine serum (FBS) (F8318, Sigma–Aldrich). The expression plasmids encoding wild-type *SSFA2* (pENTER-Flag-WT-SSFA2) were constructed by Vigene Biosciences (Jinan, China), and the mutant plasmids of *SSFA2* were generated by the Mut Express II Fast Mutagenesis Kit V2 (C214-01, Vazyme) following the instructions. The GSTM3 plasmids were synthesized and cloned into pCMV-MCS-Myc by Origene (Rockville, USA).

### LC–MS/MS analysis

Protein mixtures including SSFA2 and its interacting proteins were pulled down using the SSFA2 primary antibody by immunoprecipitation (IP) from total proteins extracted from human testes. The sample preparations and liquid chromatography–mass spectrometry/mass spectrometry (LC–MS/MS) analysis were then conducted by Hangzhou Jingjie Biotechnology Co., Ltd. (Hangzhou, China) according to standard methods, including in-gel digestion, LC–MS/MS analysis, and data processing.

### Western blotting and coimmunoprecipitation (Co-IP)

Total proteins were extracted using RIPA lysis buffer (P0013C, Beyotime) supplemented with Halt™ Protease Inhibitor Cocktail (78,425, Thermo Fisher Scientific). Samples were mixed with SDS Sample loading buffer (P0015, Beyotime) and boiled for 10 min, and then separated by electrophoresis in 7.5% or 12% SDS-PAGE gels. Subsequently, the proteins were blotted onto PVDF membranes (Millipore, Boston, USA). After an incubation with TBST containing 5% milk for 1 h, the membranes were incubated with primary antibody and horseradish peroxidase (HRP)-conjugated secondary antibodies diluted in TBST containing 5% milk. Chemiluminescence with ECL chemical substrate (WBKLS0100, Millipore) was applied for immunoblot analyses. For Western blotting, the following antibodies were used: anti-SSFA2 (1:1000; 14,157-1-AP, Proteintech); anti-Flag (1:2000; TA-05, ZSGB-Bio); anti-α-Tubulin (1:5000; ab52866, Abcam); anti-GAPDH (1:5000; ab ab8245, Abcam); HRP-conjugated Affinipure Goat Anti-Rabbit IgG (1:10000; SA00001-2, Proteintech); HRP-conjugated Affinipure Goat Anti-Mouse IgG (1:10000; SA00001-1, Proteintech).

For coimmunoprecipitation, samples were lysed in RIPA buffer (P0013C, Beyotime) supplemented with Halt™ Protease Inhibitor Cocktail (78,425, Thermo Fisher Scientific). Next, extracted total proteins were incubated with target antibodies overnight at 4 °C. Protein A/G magnetic beads (B23201, Bimake) were added to each sample and incubated for 1 hour at room temperature. After being washed three times and resuspended with 1 × PBS, the coimmunoprecipitated proteins were eluted with standard 1× SDS sample buffer and heated for 10 minutes at 95 °C. Finally, the proteins analyzed by immunoblotting as indicated. For Co-IP, the following antibodies were used: anti-SSFA2 (14157-1-AP, Proteintech); anti-F-Actin (1:1000; ab205, Abcam); anti-GSTM3 (1:1000; 67,634-1-Ig, Proteintech); anti-Flag (1:2000; TA-05, ZSGB-Bio); anti-Myc (1:2000; TA-01, ZSGB-Bio); Anti-rabbit IgG for IP Nano-secondary antibody (HRP) (1:10000; NBI01H, NBbiolab); Anti-mouse IgG for IP Nano-secondary antibody (HRP) (1:10000; NBI02H, NBbiolab).

### STA-PUT velocity sedimentation

Spermatogenic cells were obtained through cell density-gradient centrifugation using the STA-PUT velocity sedimentation method as previously described [18].

### Statistical analysis

GraphPad Prism 9.0 software was used for statistical analysis. All data are shown as the means ± standard errors of the means (SEMs). Statistical significance between two groups was calculated using an unpaired, parametric, two-sided Student’s *t* test. Statistical significance was set at *P* < 0.05.

## Results

### Clinical data

A 29-year-old man was recruited for our study who was diagnosed with 7 years of primary infertility at the West China Second University Hospital. Somatic cell karyotype (46, XY) (Supplemental Fig. 1), bilateral testicular size, secondary sex characteristics and hormone levels were normal (Supplementary Table SI). Semen analysis was performed in the clinical laboratories according to WHO guidelines [19]. The results of semen analysis are shown in Table 1. Remarkably, the sperm parameters determined by computer-assisted sperm analysis (CASA) showed that although the semen volume, sperm concentration and motility of spermatozoa in this patient were normal, all sperm heads were deformed, consisting of 100% round-headed acrosome-less spermatozoa. The patient was diagnosed with type I globozoospermia.

**Table 1:** Detailed semen parameters for the patient harboring *SSFA2* variant

Human subjects		Normospermic parameters
Age (years)	29	NA
Consanguinity (Yes/NO)	Yes	NA
**Semen parameters**		
Sperm volume (mL)	3	≥1.5
Sperm concentration (10^6^/mL)	60.8	≥15
Semen pH	7.3	≥7.2
Total sperm count (10^6^)	182.4	≥39
Motility sperm (%)	54.3	≥40
Progressive motility (%)	36.8	≥32
Vitality (%)	67	≥58
Normal spermatozoa (%)	0	≥4
Abnormal spermatozoa (%)	100	NA
Abnormal flagella (%)	0	NA
Abnormal head morphology (%)	100	NA
**Sperm locomotion parameters**		
Curvilinear velocity (VCL) (μm/s)	33.6	NA
Straight-line velocity (VSL) (μm/s)	19.1	NA
Average path velocity (VAP) (μm/s)	21.3	NA
Mean angular displacement (MAD) (degrees)	7.93	NA
Amplitude of lateral head displacement (ALH) (μm)	1.8	NA
Beat-cross frequency (BCF) (Hz)	5.4	NA
Linearity (LIN)	54.9	NA
Wobble (WOB, = VAP/VCL)	61.9	NA
Straightness (STR, = VSL/VAP)	84.1	NA

Lower and upper reference limits are shown according to the World Health Organization standards (WHO 2010) [20]

*NA* Not available

**Fig. 1:**
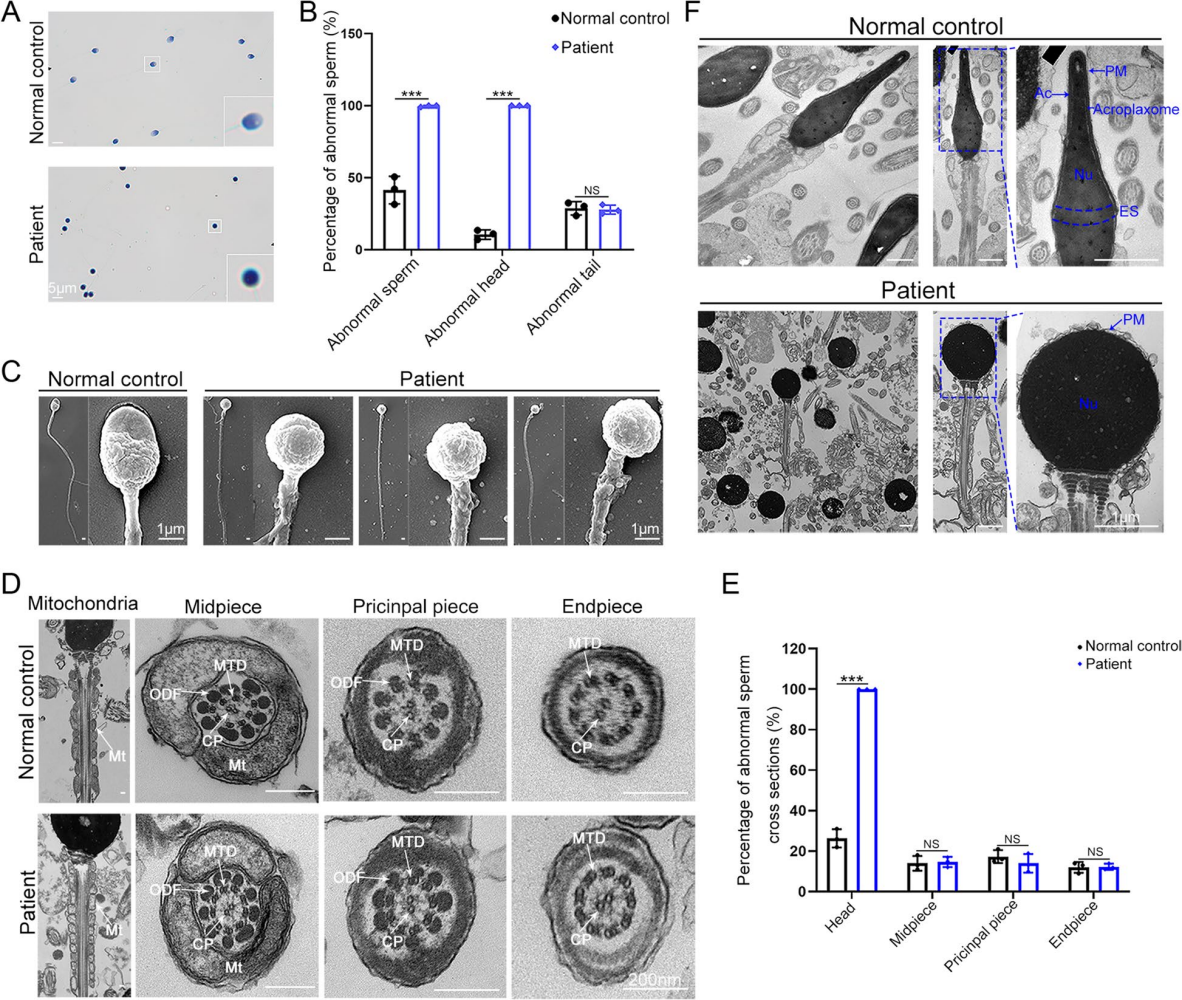
Morphology and ultrastructure of spermatozoa from the patient with globozoospermia. **A** Papanicolaou staining results showed spermatozoa from the patient with round-head and normal sperm flagella, whereas the spermatozoa from a healthy control had normal morphology. Images in the box show enlarged spermatozoa (scale bars, 5 μm). **B** Statistics on aberrant sperm in the control and the patient groups (two-sided Student’s t test; error bars, mean ± SEM. NS, *P* > 0.05; ***, *P* < 0.0005). **C** Scanning electron microscopy (SEM) micrograph showing the ultrastructural defects of sperm acrosomes in the patient (scale bars, 1 μm). **D** Transmission electron microscopy (TEM) analysis of sperm from the patient and a healthy control showed that the sperm flagella in this patient were normal and intact. Mt, mitochondria; MTD, peripheral microtubule doublets; ODF, outer dense fibers; CP, central pair (scale bars, 200 nm). **E** Statistics on the ultrastructure of sperm cross sections in controls and patients (two-sided Student’s t test; error bars, mean ± SEM. NS, *P* > 0.05; ***, *P* < 0.0005). **F** A comparative TEM view of sperm heads from the patient and a healthy control. The dotted box denotes the enlarged view of the sperm head and shows the following: compared to normal sperm heads with intact plasma membranes, acrosomes, equatorial segments and nuclei, only plasma membranes and nuclei were observed in the sperm heads of the patient. PM, plasma membrane; ES, equatorial segment; Ac, acrosome; Nu, nucleus (scale bars, 1 μm)

An evaluation of sperm morphology using Papanicolaou staining revealed abnormal sperm head development but normal sperm flagella (Fig. 1A and B). To further define the morphology of the patient’s spermatozoa, SEM was employed and exhibited aberrant sperm head morphology (Fig. 1C). To investigate whether the ultrastructure of sperm was damaged, we used TEM on this affected individual. The results showed that compared with healthy control sperm, the patient’s sperm flagella displayed a regular classic “9 + 2” structure and well-arranged mitochondria (Fig. 1D and E). The sperm heads were all round, with only a layer of plasma membrane covering them. Neither the acrosome nor the acroplaxome, which is a structure found between the acrosomal membrane and the nuclear membrane that anchors the acrosome to the nucleus during shaping of the spermatid head [21], was observed in the patient (Fig. 1F). The equatorial segment, a unique membranous structure in the middle of the sperm head that has a rather important function for fertilization [22], was also missing (Fig. 1F). Taken together, these observations suggested that this patient suffered from serious globozoospermia that further caused infertility.

### A pathogenic variant in *SSFA2* identified in the globozoospermia patient

**Fig. 2:**
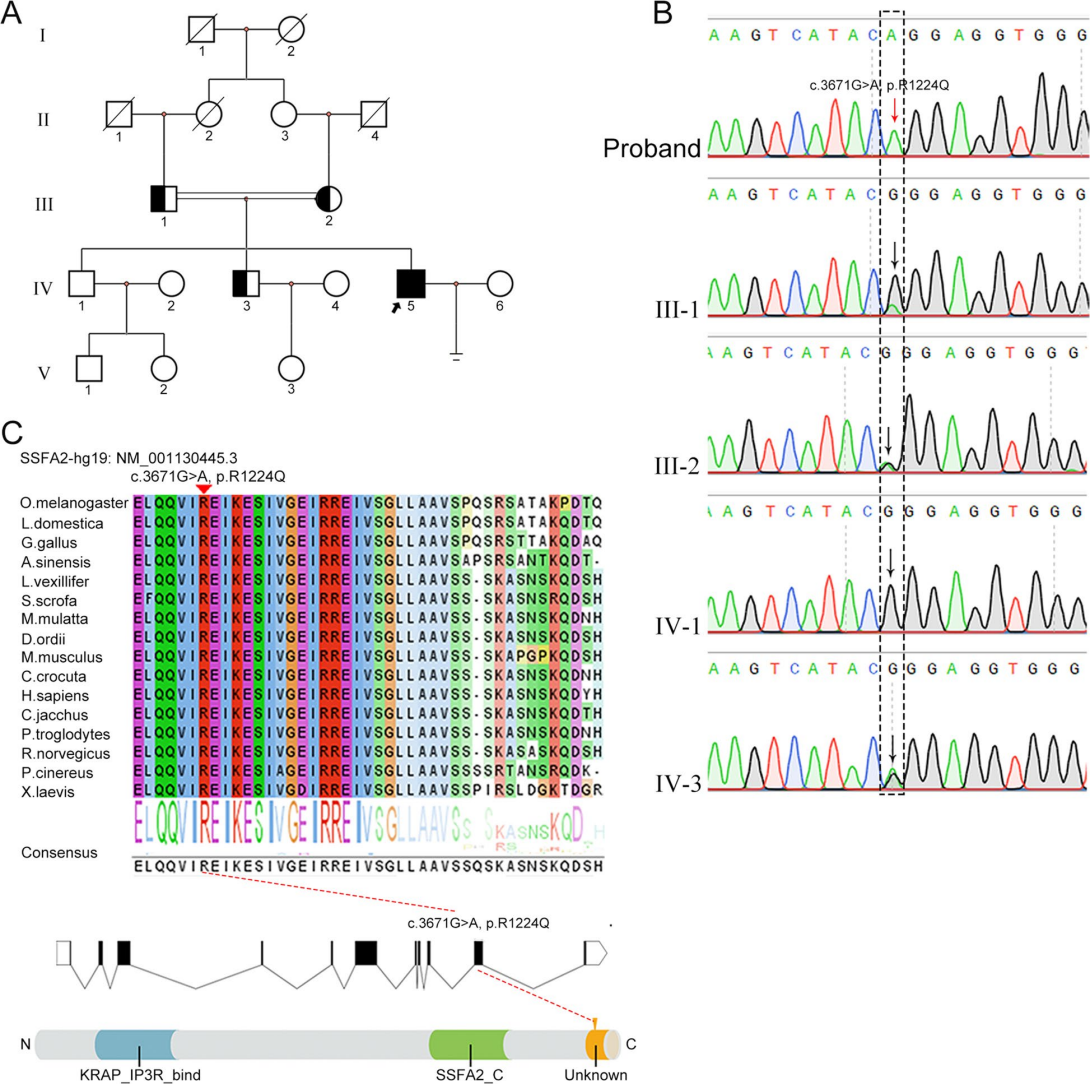
The homozygous missense variant in *SSFA2* identified in the patient. **A** Pedigree of the globozoospermia patient from a consanguineous family, with the arrow pointing to the proband. **B** Sanger sequencing verification of the patient carrying the homozygous c.3671G > A variant in *SSFA2*. The homozygotes are indicated by red arrows, and the heterozygotes are indicated by black arrows. **C** The affected amino acid residue is quite conserved across species and located in an unknown domain. Red arrows indicate the position of the missense variant. Domains of SSFA2. One KRAP_IP3R_bind domain (residues H148-T298), one SSFA2_C domain (residues T864-L1047) and one unknown domain (residues E1209-S1247) were identified

In this study, we screened for gene variants in infertile males through WES. Consequently, a novel deleterious *SSFA2* missense variant (c.3671G > A) was suggested to be the genetic cause of globozoospermia for this patient through bioinformatics analysis. The variant was absent or rare in public exome databases (dbSNP, ExAC, 1000Genomes project, and gnomAD) and was predicted to be potentially pathogenic according to SIFT, polyphen2, CADD, and MutationTaster (Supplementary Table SII), in keeping with it being a rare recessive pathogenic variant. Sanger sequencing in this family showed a cosegregation of genotype with the disease phenotype. His parents and sibling (IV-3) harbored a heterozygous variant of c.3671G > A, and his other sibling was not affected (Fig. 2A and B). Sanger sequencing verification of 220 healthy controls did not reveal the variant. The variant identified in the patient was located in exon 16 and altered a highly conserved amino acid in one unknown domain (residues E1209-S1247) of the SSFA2 protein (Fig. 2C).

### The deleterious effect of the *SSFA2* c.3671G > A variant on its expression

**Fig. 3:**
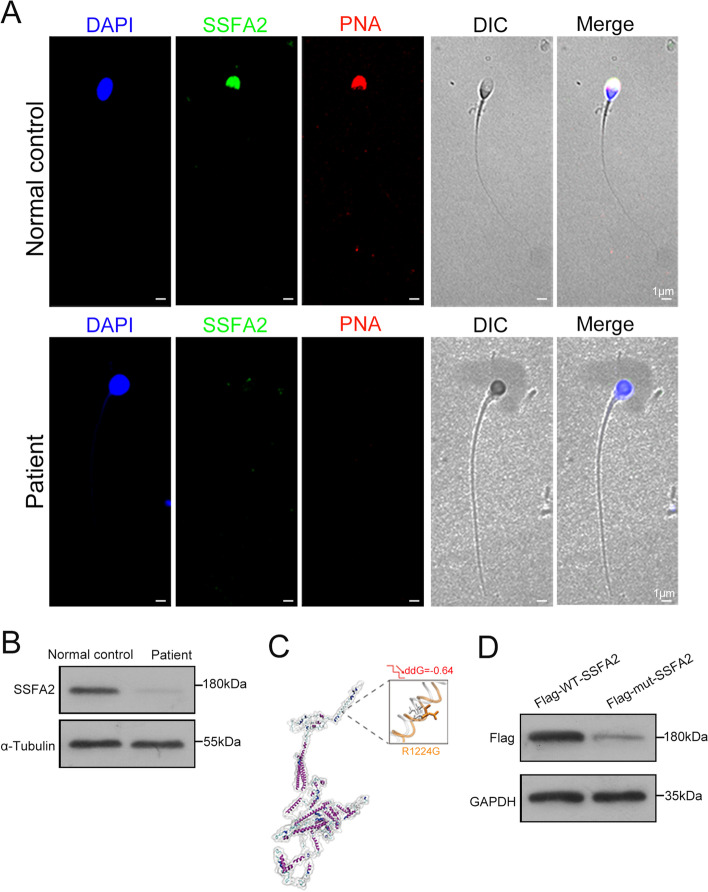
The variant of *SSFA2* is pathogenic and induced decreased protein expression of SSFA2. **A** The expression of SSFA2 in sperm was detected in healthy controls and the patient by immunofluorescence (green, SSFA2; red, PNA; blue, DAPI; scale bars, 1 μm). **B** Western blot analysis showed that SSFA2 could hardly be detected in the sperm lysates compared with the healthy control. **C** Three-dimensional structural model of SSFA2. The close-up views of the structural superposition of SSFA2-WT (white) and the corresponding variant R1224Q (orange) are displayed with a transparent new cartoon representation. The R1224 (white) and Q1224 (orange) residues are shown with Licorice representation. The change in the Gibbs free-energy gap (ddG) and the stability upon mutation are also indicated. **D** Wild-type and mutant plasmids of *SSFA2* were constructed, and protein expression was detected in HEK-293 T cells

To explore the adverse effects of the c.3671G > A variant on the expression of SSFA2, we first investigated the distribution of SSFA2 in sperm cells from the patient and healthy controls by immunofluorescence analysis. The results showed that SSFA2 was expressed in the acrosome from normal spermatozoa and merged with the acrosome marker peanut agglutinin-lectin (PNA). However, SSFA2 was not detected in the patient’s sperm (Fig. 3A). Expectedly, the expression of SSFA2 was almost undetectable in the patient’s sperm by western blot analysis (Fig. 3B). These results implied that SSFA2 plays a vital role in spermatogenesis. Subsequently, immunofluorescence staining of human testis sections showed that SSFA2 expression was obvious in different spermatogenic cells (Supplemental Fig. 2A). To further understand the localization of SSFA2 in different steps of sperm development, the STA-PUT velocity sedimentation method was used to isolate different stages of germ cells in human testes. Specifically, the SSFA2 protein was mainly distributed in the cytoplasm of spermatogonia. With the morphological transformation of spermatids, SSFA2 gradually translocated from the cytoplasm to the sperm acrosome (Supplemental Fig. 2B). In short, these results indicated that SSFA2 is essential for the development and maintenance of sperm acrosomes and that this homozygous missense variant in *SSFA2* seriously affects the expression of SSFA2.

To uncover the molecular mechanism of the c.3671G > A variant involved in SSFA2 expression, we initially predicted the conformational changes of the mutant SSFA2 by the RoseTTAFold web tool (https://robetta.bakerlab.org/submit.php) and the I-Mutant server (http://gpcr.biocomp.unibo.it/cgi/predictors/I-Mutant3.0/I-Mutant3.0.cgi). The molecular simulation showed that the Gibbs free-energy gap (ddG) and stability of mutant SSFA2 were reduced compared to those of the wild type (Fig. 3C). Next, for the analysis of mutant proteins, we established eukaryotic expression vectors of wild-type (Flag-WT-*SSFA2*) and mutant *SSFA2* (Flag-mut-*SSFA2*) including the variant site, and then transfected them into HEK293T cells. Markedly reduced protein levels of SSFA2 were observed in cells overexpressing the Flag-mut-*SSFA2* plasmid compared to cells overexpressing the Flag-WT-*SSFA2* plasmid by western blot analysis (Fig. 3D). Collectively, these results suggested that this missense variant of c.3671G > A in *SSFA2* significantly reduced SSFA2 expression and impaired the development of the sperm acrosome and further led to the globozoospermia phenotype.

### SSFA2 interacted with GSTM3 and Actin

**Fig. 4:**
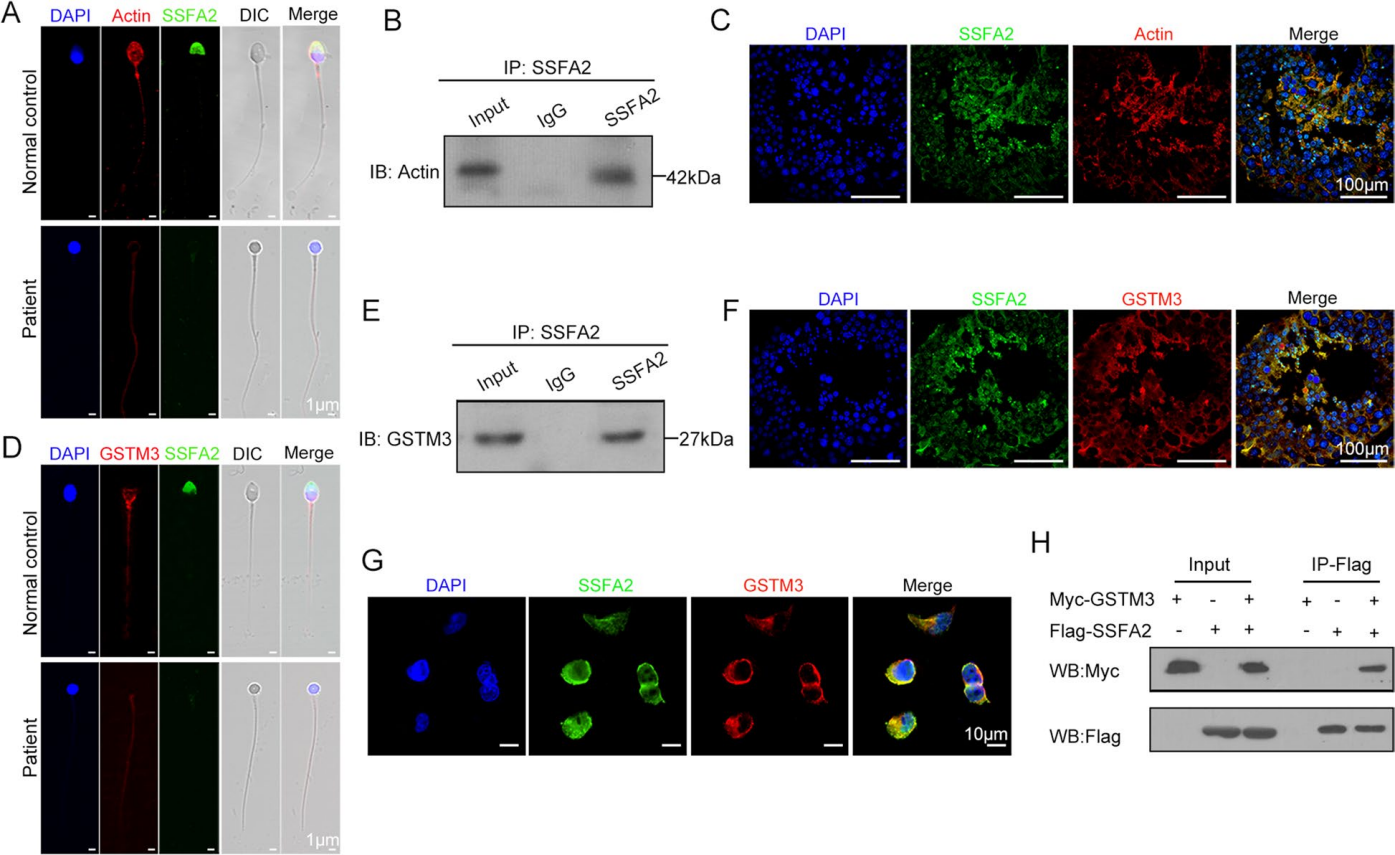
SSFA2 interacted with GSTM3 and Actin during spermatogenesis. **A** In a healthy control, Actin was located in the head and tail, and SSFA2 was located in the acrosome. Actin and SSFA2 signals were barely detectable in the sperm head of the patient (green, SSFA2; red, Actin; blue, DAPI; scale bars, 1 μm). **B** The interaction between SSFA2 and Actin was identified by coimmunoprecipitation. **C** Colocalization of SSFA2 and Actin was examined by immunofluorescence in testes from healthy controls (green, SSFA2; red, Actin; blue, DAPI; scale bars, 100 μm). **D** In spermatozoa from a healthy control, GSTM3 localized to the tail and equatorial plate and colocalized with SSFA2 on the equatorial plate, but only GSTM3 localized to the flagella was detected in the patient sperm (green, SSFA2; red, GSTM3; blue, DAPI; scale bars, 1 μm). **E** The interaction between SSFA2 and GSTM3 was identified by coimmunoprecipitation. **F** Colocalization of SSFA2 and GSTM3 was examined by immunofluorescence in testes from healthy controls (green, SSFA2; red, GSTM3; blue, DAPI; scale bars, 100 μm). **G**, **H** In vitro experiments demonstrated the interaction between SSFA2 and GSTM3. The Myc-WT-GSTM3 plasmid and the Flag-WT-SSFA2 plasmid were cotransfected into the HEK293T cell line, and immunofluorescence revealed that GSTM3 and SSFA2 colocalized in the cytoplasm. Further co-IP analysis also verified their interaction (green, SSFA2; red, GSTM3; blue, DAPI; scale bars, 100 μm)

To further explore the potential mechanism of SSFA2 in spermatogenesis, immunoprecipitation of SSFA2 from normal human testes followed by LC–MS/MS analysis was adopted and revealed 58 interactors of SSFA2. Among them, GSTM3 and Actin were relatively highly abundant. Previous research showed that the N-terminal region of SSFA2 may interact with IP3 receptors (IP3Rs), while the C-terminal region may interact with Actin. SSFA2 tethers IP3Rs to Actin alongside sites where store-operated Ca^2+^ entry occurs, licensing them to evoke cytosolic Ca^2+^ signals [14, 23]. Immunofluorescence staining showed that SSFA2 and Actin colocalized in the sperm acrosome (Fig. 4A). We further confirmed their interactions by Co-IP and immunofluorescence analyses in human testes (Fig. 4B and C). GSTM3 is abundantly expressed in human testes and is involved in sperm-zona pellucida binding events in which GSTM3 binds to ZP4 during the first steps of gamete recognition to allow fertilization to occur [24–26]. GSTM3, located in the tail and equatorial subdomain, has been recently established as a fertility and cryotolerance biomarker in boar sperm [27–29]. As expected, GSTM3 was also located on the tail and equatorial plate of humans and colocalized with SSFA2 on the equatorial plate of sperm (Fig. 4D). Co-IP and immunofluorescence analyses also demonstrated the interactions in human testis (Fig. 4E and F). Furthermore, the Myc-WT-*GSTM3* plasmid and the Flag-WT-*SSFA2* plasmid were cotransfected into the HEK293T cell line, and immunofluorescence staining showed that they were colocalized in the cytoplasm (Fig. 4G). Co-IP also proved that GSTM3 and SSFA2 can interact in vitro (Fig. 4H).

### Performing ICSI on the patient had a poor outcome

**Fig. 5:**
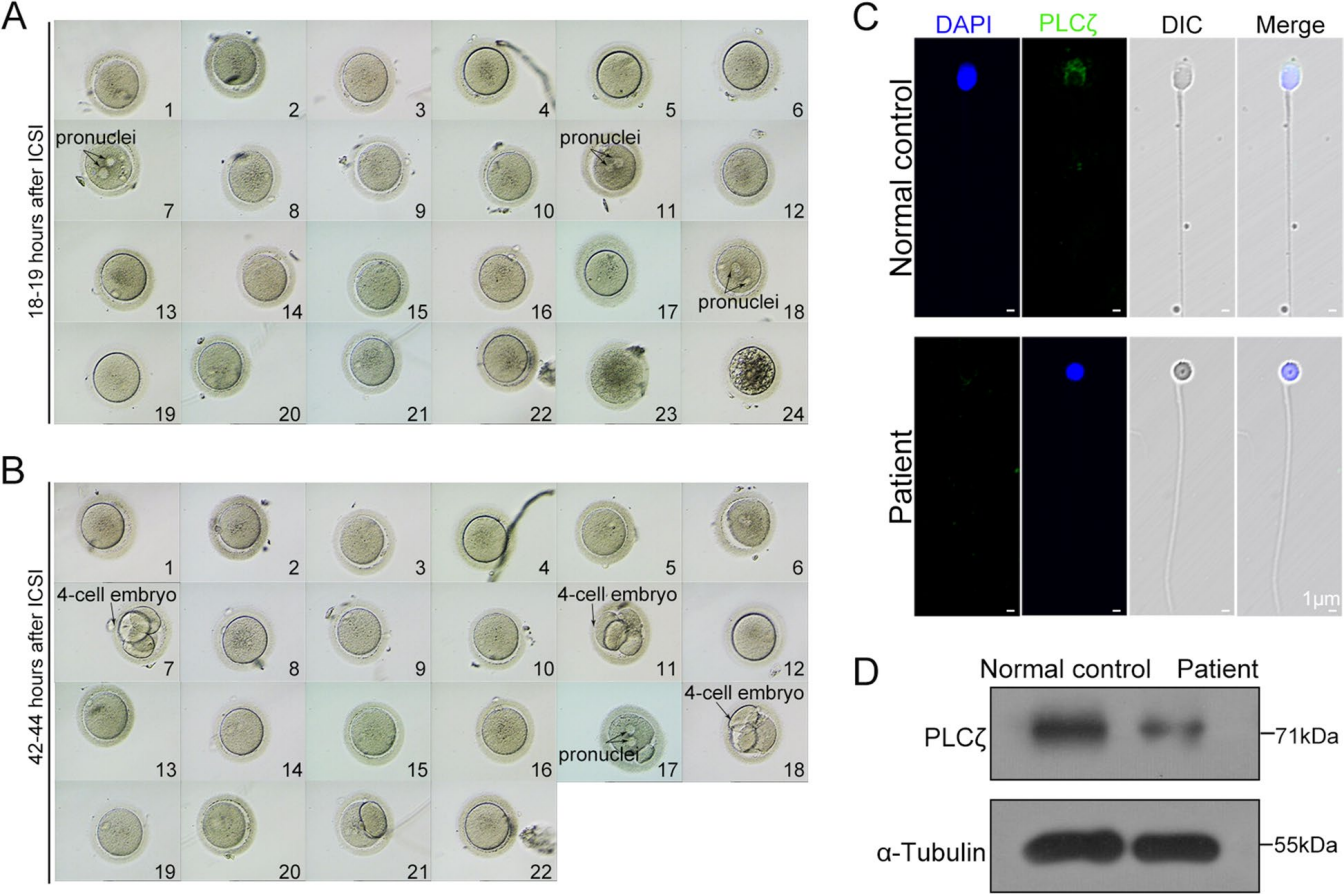
Clinical features of the couple undergoing ICSI treatment. **A** Eighteen to 19 hours after regular ICSI, the majority of eggs failed to be fertilized by microscopy, and the black arrow indicates pronuclei. **B** Forty-two to 44 hours after regular ICSI, three zygotes developed into three poor-quality 4-cell embryos. **C**, **D** Immunofluorescence and western blot analysis showed that PLCζ expression in patient sperm was significantly reduced compared to the healthy control (green, PLCζ; blue, DAPI; scale bars, 1 μm)

Primarily, a regular ICSI cycle was attempted for the couple, and an informed consent form was signed for the ICSI procedure. The basal hormone data of the patient’s wife were regular (Table 2). Ovulation induction was performed, and the long protocol is presented in Table 2. Consequently, 24 mature oocytes were aspirated during follicular puncture, and the ejaculated sperm from the patient were injected into the 24 oocytes for the ICSI cycle. As shown in Table 2, the cycle achieved 16.7% fertilization; apparently, the sperm obtained from this patient failed to activate most MII oocytes (Fig. 5A). Three of the four embryos reached the cleavage stages (Fig. 5B). Unfortunately, none of them developed into high-quality embryos for transfer. The outcome of regular ICSI treatment suggested that the patient’s sperm defect caused oocyte activation failure. Phospholipase C zeta (PLCζ), an important sperm factor, is widely considered to be one of the physiological stimuli responsible for the generation of Ca^2+^ oscillations that induce egg activation and early embryonic development [30, 31]. It has been shown that defects in PLCζ in infertile male patients lead to failure of egg activation following ICSI [32–39]. Therefore, we examined the expression of PLCζ in the patient’s sperm. Immunofluorescence staining showed that the expression of PLCζ in the patient’s sperm was significantly lower than that in healthy controls (Fig. 5C). This observation was further confirmed by western blotting analysis (Fig. 5D).

**Table 2:** Clinical features of the patient with ICSI treatment

Male age (y)	29
Female age (y)	26
Length of primary infertility history (y)	6
BMI (kg/m^2^)	21.63
Basal hormones	
FSH (IU/L)	5.7
LH (IU/L)	6.3
E2 (pg/mL)	131
PRL (ng/ml)	475.5
Prog (ng/ml)	1.0
Testo (ng/ml)	0.4
Cycle 1	
Protocol	Long
E2 level on the trigger day (pg/mL)	3383
No. of follicles ≥14 mm on the trigger day	16
No. of follicles ≥18 mm on the trigger day	3
No. of oocytes retrieved	30
No. of mature oocytes	24
ICSI progress	
Oocytes injected	24
Fertilization rate (%)	16.7% (4)
Cleavage Rate (%)	12.5% (3)
6 cell formation rate (%)	0
8 cell formation rate (%)	0
Cycle 2	
Protocol	Long
E2 level on the trigger day (pg/mL)	3845
No. of follicles ≥12 mm on the trigger day	19
No. of oocytes retrieved	10
No. of mature oocytes	10
AOA-ICSI progress	
Oocytes injected	10
Fertilization rate (%)	100% (10)
Cleavage Rate (%)	100% (10)
6 cell formation rate (%)	80% (8)
8 cell formation rate (%)	40% (4)

A previous study revealed that PLCζ defect-associated oocyte activation failure was rescued by ICSI with AOA [40]. After the first failed ICSI attempt, we performed AOA with a calcium ionophore (A23187) after the second ICSI attempt. Delightfully, in the AOA-ICSI cycle, 10 MII oocytes were injected with sperm from the patient, all of them achieved successful normal fertilization after AOA, and eight transferable embryos were obtained (with five good-quality embryos) (Table 2). After embryo selection, we transferred embryos (one eight-cell embryo) on Day 3 and achieved pregnancy, and a healthy baby was born to the couple. These results suggest that *SSFA2* variant-associated infertility could be successfully rescued by ICSI with AOA.

## Discussion

In the present study, we identified a novel globozoospermia causative gene, *SSFA2,* in an infertile patient. Light microscopy results showed severe abnormalities in the morphology and ultrastructure of the patient’s sperm head. We confirmed the harmfulness of missense variant in *SSFA2* with both a bioinformatic analysis and an in vitro expression study. Notably, the expression of SSFA2 in human testes and in the different germ cell types during spermiogenesis suggests the considerable role of SSFA2 in the development of the sperm acrosome. The key role of SSFA2 in acrosome formation and oocyte activation was established in the current study. Due to oocyte activation deficiency, regular ICSI for the patient completely failed. Reassuringly, AOA after ICSI successfully overcame oocyte activation failure, and a healthy baby was born to the couple.

Previous research has shown that the total motility, progressive motility and normal morphology in both types of globozoospermia samples were lower than those in normozoospermic controls [8, 41]. Intriguingly, the semen parameters of this patient carrying the homozygous c.3671G > A variant of *SSFA2* were normal except for the morphology of the sperm head, including the semen volume, concentration, sperm motility, and sperm tail morphology, which implied an intriguing possibility that SSFA2 plays a specific role in the acrosome and fertilization and not the other way around. Herein, we showed that SSFA2 can interact with Actin and GSTM3 during spermatogenesis. A recent study showed that GSTM3 is located in the tail and equatorial subdomain of the head of boar sperm [29]. Similarly, GSTM3 was observed in the human sperm tail and equatorial subdomain and was partially colocalized with SSFA2. In mammalian sperm, Actin is present in the equatorial plate, posterior acrosome area and tail in its monomeric form, as well as filamentous Actin [42–47], which is essential for acrosome formation, sperm capacitation and the acrosome reaction [48–51]. We therefore conclude that SSFA2 interacts with GSTM3 on the equatorial plate and Actin in the acrosome to promote and maintain acrosome formation during spermatogenesis and does not participate in sperm flagellogenesis and motility regulation. How the separate regulation of acrosome formation and flagellogenesis was achieved is an open question that deserves further investigation.

Since the introduction of ICSI, globozoospermic patients have undergone therapeutic treatment, but the prognosis of these patients is still unsatisfactory. Recent studies suggest that ICSI with AOA has a high fertilization rate in globozoospermic patients with defective PLCζ [52–54]. Our study found that the expression of PLCζ in this globozoospermic patient carrying the *SSFA2* variant was significantly reduced. In the ICSI-AOA cycle, the rates of normal fertilization (2PN) were significantly increased compared with those in the regular ICSI cycle (100% vs. 16.7%), and a healthy baby was born after transferring one good-quality embryo, suggesting that ICSI with AOA may be a viable treatment for patients carrying the *SSFA2* variant.

In mammals, fertilization triggers a pathway that induces cytosolic calcium Ca^2+^ oscillations that persist for several hours [55] and are the common signal of oocyte activation. Previous studies have shown that PLCζ plays an important role in egg activation, but eggs fertilized with PLCζ knockout sperm still exhibited 3–4 Ca^2+^ oscillations in total [56]. Such observations suggest that sperm contain other factors with Ca^2+^ releasing activity. Tr-kit [57], citrate synthase [58] or PAWP [59] were found to have a contributory function in Ca^2+^ release at oocyte activation, while none of them has been shown to be directly involved in IP3-mediated Ca^2+^ release. A recent study indicated that SSFA2 is directly involved in IP3-mediated Ca^2+^ release in HEK cells and HeLa cells, suggesting that SSFA2 in sperm may also be involved in IP3-mediated Ca^2+^ oscillations upon oocyte activation. The detailed mechanism and the relationship between SSFA2 and PLCζ in oocyte activation warrant further investigation.

In conclusion, we have identified *SSFA2* as a novel causative gene for male infertility associated with globozoospermia, which interacts with Actin and GSTM3 and is important for the development of sperm acrosomes and the activation of oocytes. More excitingly, ICSI with AOA successfully overcame the patient’s infertility. Our study will help to evaluate globozoospermia with an unknown etiology. In the future, a larger globozoospermic cohort needs to be studied to identify pathogenic variants of *SSFA2* and unknown genes accounting for globozoospermia, which will assist in the accurate diagnosis and clinical management of globozoospermia patients.

## Conclusions

Collectively, our findings identified loss-of-function SSFA2 as a new factor contributing to male infertility with globozoospermia, and revealed a role for this gene in regulating acrosome formation during spermatogenesis. Of particular concern is the good prognosis of the patient with *SSFA2* variant after AOA-ICSI treatment, indicating that the SSFA2 gene associated with evoked cytosolic Ca^2+^ signals in HEK cells and HeLa cells may provide a clue for insights into oocyte activation failure and clinical strategies for patients with globozoospermia.

## Supplementary Information

**Additional file 1: Supplemental Figure S1.** Karyotype analysis of the patient: normal male karyotype 46, XY. **Supplemental Figure S2.** The expression pattern of SSFA2 in humans. (A) SSFA2 showed higher expression in early and late spermatids, whereas it was also expressed in spermatogonia and spermatocytes (green, SSFA2; red, PNA; blue, DAPI; scale bars, 100 μm). (B) The expression pattern of SSFA2 in spermatogenic cells at different stages. Sa1-Sb1, round spermatid. Sb2-Sd2, elongating spermatids (green, SSFA2; red, PNA; blue, DAPI; scale bars, 1 μm). **Supplemental Table SI.** Basal blood hormone levels. **Supplemental Table SII.** Analysis of *SSFA2* Variant in the patient.

## Data Availability

The data underlying this article will be shared on reasonable request to the corresponding author.

## Abbreviations

WES: Whole-exome sequencing
LC–MS/MS: Liquid chromatography–mass spectrometry/mass spectrometry
Co-IP: Coimmunoprecipitation
ICSI: Intracytoplasmic sperm injection
AOA: Artificial oocyte activation
