# Supplementary material for: A loss-of-function variant in SSFA2 causes male infertility with globozoospermia and failed oocyte activation
Source: Reprod Biol Endocrinol. 2022 Jul 14;20:103. doi: 10.1186/s12958-022-00976-5 (PMC9281110; doi:10.1186/s12958-022-00976-5)
Supplement: Supplementary file 1 — Additional file 1: Supplemental Figure S1. Karyotype analysis of the patient: normal male karyotype 46, XY. Supplemental Figure S2. The expression pattern of SSFA2 in humans. (A) SSFA2 showed higher expression in early and late spermatids, whereas it was also expressed in spermatogonia and spermatocytes (green, SSFA2; red, PNA; blue, DAPI; scale bars, 100 μm). (B) The expression pattern of SSFA2 in spermatogenic cells at different stages. Sa1-Sb1, round spermatid. Sb2-Sd2, elongating spermatids (green, SSFA2; red, PNA; blue, DAPI; scale bars, 1 μm). Supplemental Table SI. Basal blood hormone levels. Supplemental Table SII. Analysis of SSFA2 Variant in the patient. [file 12958_2022_976_MOESM1_ESM.pdf]

A loss-of-function variant in *SSFA2* causes male infertility with globozoospermia and failed oocyte activation

Huang et al.

Supplementary information

Supplementary Figures

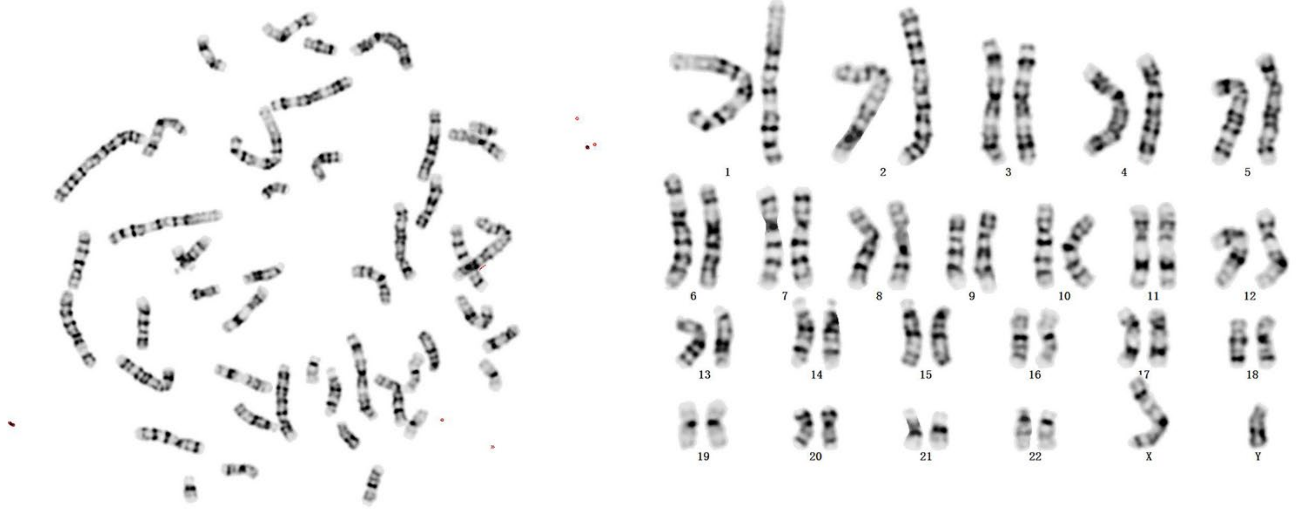

Supplemental Figure S1. Karyotype analysis of the patient: normal male karyotype 46, XY.

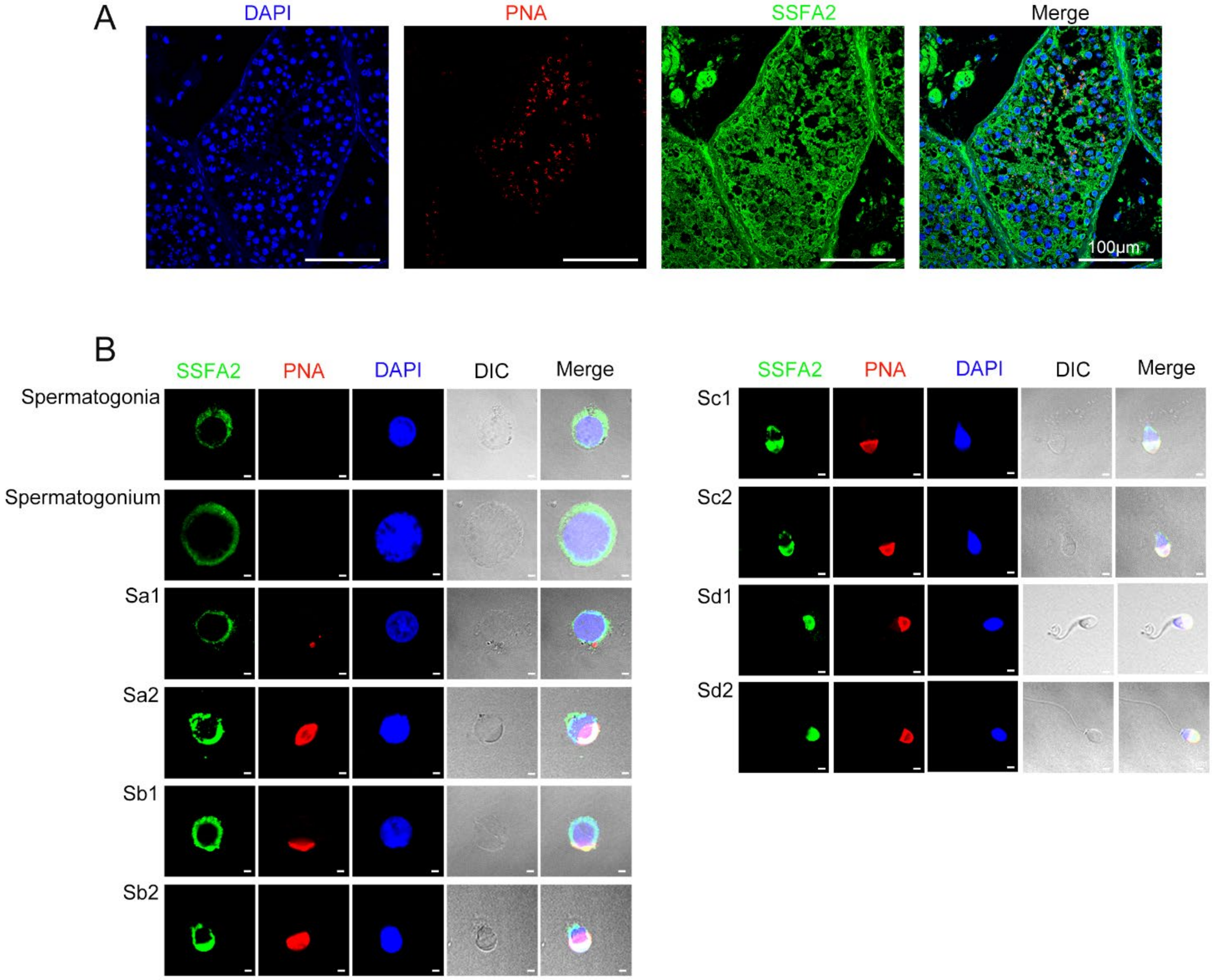

Supplemental Figure S2. The expression pattern of SSFA2 in humans.

(**A**) SSFA2 showed higher expression in early and late spermatids, whereas it was also expressed in spermatogonia and spermatocytes (green, SSFA2; red, PNA; blue, DAPI; scale bars, 100 μm). (**B**) The expression pattern of SSFA2 in spermatogenic cells at different stages. Sa1-Sb1, round spermatid. Sb2-Sd2, elongating spermatids (green, SSFA2; red, PNA; blue, DAPI; scale bars, 1 μm).

Supplementary tables

Supplemental Table SI. Basal blood hormone levels

Hormone	Baseline	Reference Range
E2 (pmol/L)	41.0	0-156
FSH (mIU/mL)	3.2	1.5-12.4
LH (mIU/mL)	2.2	1.7-8.8
PRL (mIU/L)	283.4	86-324
Testosterone (nmol/L)	14.2	8.6-29.0

Supplemental Table SII. Analysis of *SSFA2* Variant in the patient

Phenotype	Globozoospermia
cDNA mutation	c.3671G>A
Genotype	Homozygous
Protein alteration	p.R1224Q
Mutation type	Missense
dbSNP	0
Allele frequency in ExAC	0
1000G project	0
gnomAD	0.00000427
Function prediction	
SIFT	Deleterious
PolyPhen-2	Probably damaging
CADD_phred	24.1[a]
Mutation Taster	Disease causing

*The GenBank accession number for *SSFA2*: NM_001130445.3

[a] The points>10 means deleterious
